# Cook County Hospital

**Published:** 1869-11

**Authors:** Curtis T. Fenn


### Hospital Reports.

ARTICLE I.— *Cook County Hospital.* Reported by CURTIS T. FENN, M.D.

THE following is a brief summary of reports for the month of September, 1869:

There were in the Medical Department, at the beginning of the month, 38; admitted during the month, 74; discharged cured, 36; discharged relieved, 5; sent to the County House, 2; absconded, 1; died, 16; remaining, 52.

In the Surgical Department at the beginning of the month, 33; admitted, 14; discharged cured, 20; discharged relieved, 1; discharged incurable, 1; died, 2; remaining, 23.

In the Gynæcological Department at the beginning of the month, 19; admitted, 17; discharged cured, 9; discharged relieved, 2; absconded, 1; died, 1; remaining, 23; births, 16.

In the Ophthalmic and Aural Department at the beginning of the month, 17; admitted, 5; discharged cured, 2; discharged relieved, 2; absconded, 1; remaining, 19.

Total at the beginning of the month, 107; admitted, 112; discharged cured, 67; discharged relieved, 10; discharged incurable, 1; absconded, 3; sent to County House, 2; died, 19; remaining, 117.

There were in the Gynæcological Department at the beginning of September, 1868, 13; admitted, 15; discharged cured, 3; at own request, 1; absconded, 1; died, 1; remaining, 23; births, 8.

An increase equal at least to that, has occurred in each of the other departments. Two new wards have been provided, lately, by alterations in the basement, the only extension possible at present. There is reason to believe that even with double the room now afforded, needy sufferers would soon be in excess of places to receive them.

Of course the labor of the attendants is increasing. The house physician and his two assistants, carry a great responsibility, with credit to all concerned, be it said. Registration, diet lists, prescriptions, surgical dressings, midwifery, *post mortems*, monthly reports, run up a startling aggregate of work each month. If the hospital did no more than complete the education of its internes, it would fill a good mission. The breadth of experience, the coolness and efficiency acquired by contact day and night for a year and a half, with such an accumulation of humanity in mortal combat, is a proper finish to a medical education. The hospital imparts more than lectures, or books, or travel. The interne is taken into the closest intimacy with suffering and death. He comes face to face with what years of private practice will but slowly unfold. Experience, if waited for, generally arrives so late as to serve but a brief space, when other knowledge with which it ought to be joined, has faded from memory. Some things are gained by patient, slow, laborious plodding. But much that a medical man must ever hold as the highest and best attainment, is reached intuitively. There is a capacity which amounts to genius, in the majority of those who adopt the study of medicine. It seeks recognition in affinity for clinics and autopsies. This eagerness is not morbid curiosity. It is enthusiasm urging them to come eye to eye with their work. It is an exchange of civilities between nature and her votaries. With his five senses, under favoring circumstances, one will intuitively arrive at clearer perceptions of truth than wordy sketches or diagrams, or models, will ever convey. The student desires the presence of nature. This enthusiasm ought to be gratified early. In its track books become illuminated. Readiness to recognize facts: focusing of all the intelligence upon objects, is a proof of genius. Facts rob theories of undue proportions. The hospital, in a word, is a great teacher. Its grand object-lessons never become stale. The truths they utter flash upon the mind at once. Teachable souls are there admitted to the secret of the Almighty.

We are never surprised, therefore, that a large number of graduates annually seek the appointment of interne. It is to perfect a foundation for future study — acquaintance with actual, tangible facts, such as appeal to us in pallid faces and vacant cots.

The house physician is now required to keep a reference file for each ward. Every important case is entered upon a slip of paper; previous and current history noted, and this slip is filed. When removed, it is pasted in a scrap-book. This tax upon him is one of the most important in the way of discipline, and affords a valuable accession to the records of the hospital.

Cliniques are again in vogue: two Medical, two Surgical, one Ophthalmic, one Gynæcological or Dermatological each week.

Prof. Johnson conducts the Medical clinique at 1.30, and Prof. Powell the Surgical clinique at 2.30, Tuesdays and Fridays.

Dr. J. H. Hildreth holds a clinique at 1.30, and either Dr. H. W. Jones or Dr. C. G. Smith at 2.30 Saturdays.

A large body of students from the different Medical Colleges are in attendance. Medical practitioners from the city and country are always ready to attend. The fund received by the sale of tickets has been large, and is appropriated towards paying current expenses. The mission of the County Hospital is a noble one, not only in regard to its influence as an educational means, but also as an institution of public charity. We bespeak for it whatever will promote the convenience of its attending and resident physicians. A small sum taken from the income of tickets annually, and appropriated to the general good, might be used to materially lighten the burden of those who are called to serve.

